# Cytokine alterations during paraneoplastic neutrophilia and leukemoid reaction in patients with advanced melanoma

**DOI:** 10.1007/s00262-022-03249-7

**Published:** 2022-07-16

**Authors:** Xin-Wen Zhang, Alexander Wald, Martin Salzmann, Niels Halama, Jessica C. Hassel

**Affiliations:** 1 Department of Dermatology and National Center for Tumor Diseases, University of Heidelberg, Im Neuenheimer Feld 460, 69120 Heidelberg, Germany; 2 Department of Oncology and National Center for Tumor Diseases, University of Heidelberg, Im Neuenheimer Feld 460, 69120 Heidelberg, Germany

**Keywords:** Melanoma, Cytokines, Neutrophils, Inflammation, Paraneoplastic leukemoid reaction

## Abstract

**Background:**

Paraneoplastic leukemoid reaction (PLR) is a rare phenomenon in metastasized melanoma associated with poor prognosis and rapid disease progression. Currently, no specific therapeutic options exist other than treating the underlying malignancy.

**Methods:**

Five cases of paraneoplastic neutrophilia in patients with advanced-stage IV melanoma were enrolled in our study. Cytokine concentrations in patients’ serum samples were analyzed before and during PLR using a multiplex cytokine array. Further, immunohistochemical staining of tumor tissue biopsied during PLR was performed.

**Results and conclusions:**

We observed a strong correlation between worsening of tumor burden and aggravation of neutrophilia. Cytokine measurements revealed an increase of proinflammatory cytokines (IL6, IFN*γ*), proangiogenic cytokines (VEGF) and immune stem cell growth factors (G-CSF) during PLR. Immunohistochemistry confirmed neutrophil infiltration of tumor tissue. The presented cytokine alterations provide a basis for further functional analysis, which is necessary for the development of targeted therapeutic approaches against PLR.

**Supplementary Information:**

The online version contains supplementary material available at 10.1007/s00262-022-03249-7.

## Introduction

Paraneoplastic leukemoid reaction (PLR) is defined as a tumor-induced leukocyte count of above 50 /nl and has been described in different tumor entities, including melanoma. Unawareness of this phenomenon may lead to unnecessary antibiotic treatment and could delay specific anti-cancer therapy [1, 2].

The precise pathological mechanisms of PLR are unclear, previous reports suggest a secretion of cytokines by tumor cells [3]. An association between elevated G-CSF [4, 5], IL6, IL8, and IL10 [6] concentrations and the occurrence of PLR was observed in case reports.

The goal of our analysis was an investigation of underlying mechanisms of PLR in cases observed at our institution.

## Methods

### Clinical data

This is a retrospective, monocenter study. We screened internal records for the occurrence of paraneoplastic neutrophilia or PLR in patients treated for metastasized melanoma at our institution. Neutrophil counts and laboratory values were measured as clinically indicated at the time of blood draw. Retrospective analyses of patient’s data and samples were approved by the ethics committee of the University Hospital Heidelberg (S-454/2015, S-207/2005).

### Cytokine measurements

**Fig. 1:**
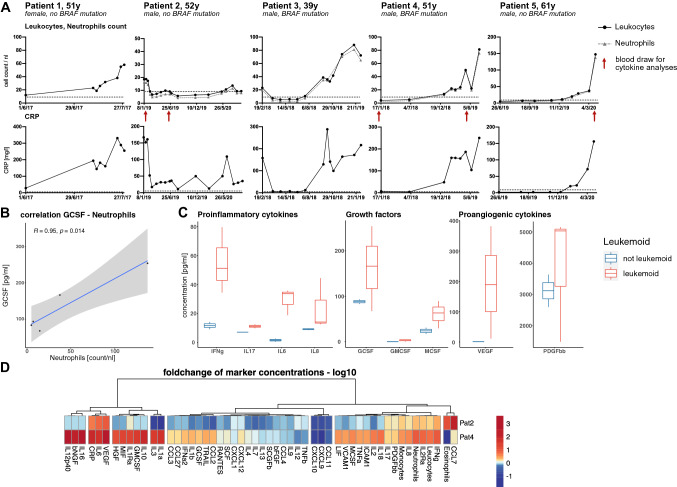
Paraneoplastic leukemoid reaction in melanoma **A** Progression of absolute leukocyte count, absolute neutrophil count (count/nl), and C-reactive protein (mg/l) during disease course. Time points of blood draw for cytokine analysis (patients 2, 4, and 5) are marked by red arrows. **B** Correlation of serum G-CSF concentration to absolute neutrophil count (*n* = 5). A significant correlation was observed (Pearson’s correlation coefficient, *R* = 0.95, *p* = 0.014). **C** Comparison of serum cytokine concentrations between not-leukemoid status and leukemoid reaction (not leukemoid: *n* = 2, leukemoid: *n* = 3). **D** Hierarchical clustering of serum cytokines and blood markers based on euclidean distance calculations. Marker concentrations are normalized as log_10_ of fold-change to baseline concentrations (*n* = 2)

Multiplex cytokine analyses were performed using a two-laser array reader system (Bio-Rad, Munich, Germany) for quantification of multiple cytokines and chemokines. Briefly, serum samples obtained at different time points during disease (Fig. 1A) were processed with respective dilutions, and calibration of investigated analytes was performed according to the manufacturer’s instruction. Bio-Plex Manager 4.1.1 was used for generation of standard curves and concentrations. Further data analyses were performed with R version 4.0.3.

### Immunohistochemistry

Paraffine-embedded histological samples of tumor tissue were stained for infiltration of CD3 and MPO expressing cells. Hematoxylin was used for counterstaining.

## Results

Within the last three years, we observed five cases of paraneoplastic neutrophilia in patients with stage-IV melanoma (Fig. S1).

Patient 1 was a 51-year-old woman immunosuppressed with azathioprine for systemic lupus erythematosus, presented with cutaneous stage-IV BRAF-wildtype melanoma in May 2017. After radiotherapy of a symptomatic bone metastasis, immunotherapy was declined until July 2017 when she presented again with massively progressive disease. She died two days after immediate administration of ipilimumab and nivolumab. At treatment onset, she reached an absolute neutrophil count (ANC) of 58/nl (7.5 × upper limit of normal ULN) accompanied by an increase in C-reactive protein (CRP) up to 331 mg/l (66xULN) (Fig. 1A). Infection was ruled out by clinical (incl. urine analysis) and radiological (CT scan of chest and abdomen) examination, indicating paraneoplastic neutrophilia. No hematological workup was possible within the short timeframe. Biopsy of a subcutaneous metastasis revealed a massive neutrophilic infiltration (Fig. S1).

Patient 2 was a 52-year-old man, with stage-IV melanoma of unknown origin, BRAF-wildtype. He first presented in January 2019 with newly diagnosed bone and soft tissue metastases. Notably, ANC was elevated at 19.4 /nl (2.5 × ULN), CRP was 166.9 mg/l (33xULN) (Fig. 1A). Infectious and hematological causes were ruled out by extensive clinical (including an esophagogastroduodeno- and colonoscopy), laboratory (including procalcitonin levels, blood cultures, tuberculosis screening, serology for hepatitis A, B, C, and HIV), and radiological examination (chest CT, abdominal MRI), bone marrow aspiration and FACS-analysis of the peripheral blood. Assuming a paraneoplastic cause for neutrophilia, immunotherapy was initiated within a clinical trial. During oncological response to immune-checkpoint-inhibition, ANC and CRP normalized without concomitant antibiotic treatment (Fig. 1A). In comparison to patient 1, tumor biopsy of patient 2 was less infiltrated with neutrophils (Fig. S1), which is in accordance with a lower absolute neutrophil count.

Patient 3, a 39-year-old man, first presented at our center in February 2018. He was diagnosed with stage-IV cutaneous melanoma, BRAF V600E, in December 2016. He had already received targeted therapy with dabrafenib/trametinib and dual checkpoint inhibition with ipilimumab and nivolumab, followed by nivolumab monotherapy until progressive disease. At first visit, ANC was 19.41 /nl (2.5xULN), CRP 158 mg/dl (31xULN) (Fig. 1A). Both values decreased to normal within four weeks after reinduction of dabrafenib and trametinib. Upon development of resistance against targeted therapy, ANC and CRP rose again, synchronously to measurable disease progression. CT and MRI imaging, clinical examinations, urine analyses, and blood cultures revealed no signs of infection. Leukemia was ruled out by FACS analysis and PCR for BCR-ABL. He did not respond to any subsequent treatment. Shortly before death, we measured an ANC of 101 /nl (13xULN) and a CRP of 223 /dl (56xULN) (Fig. 1A).

Patient 4 was a 51-year-old man, diagnosed with stage-IV cutaneous melanoma harboring a BRAF-V600E mutation. Between December 2015 and December 2018, he consecutively progressed under ipilimumab and nivolumab combination therapy, dacarbazine, and off-label treatment with avelumab, with intermittent successful re-challenge of targeted therapy between each treatment line. Elevation of ANC and CRP was first observed in January 2019 (Fig. 1A). Shortly after, CT scan confirmed progressive disease. No infectious cause of neutrophilia could be determined by repetitive clinical (incl. urine analyses and blood cultures) and radiological (incl. abdominal / chest CT, brain MRI) checkups. Overall, BRAF/MEK-inhibition was re-introduced four times, each time leading to response and decrease of ANC and CRP of short duration. On the day of death in August 2019, we noted an ANC of 129.8 /nl (17xULN) and CRP of 250.2 mg/dl (50xULN) (Fig. 1A).

Patient 5, a 61-year-old man, first presented in May 2019 with an unresectable stage-III cutaneous melanoma, BRAF wildtype, with extensive metastases of the neck. He previously received adjuvant anti-PD-1 therapy, and at progression was included in a clinical trial testing ipilimumab combined with intralesional application of a toll-like receptor agonist. After progression, he received definitive radiotherapy to the neck. In January 2020, a rising ANC and CRP were first observed with 20 /nl (2.6xULN) and 20.6 mg/l (4xULN), respectively (Fig. 1A). After developing pulmonal metastases, palliative chemotherapy with dacarbazine was initiated. At this point, ANC had reached 147 /nl (19xULN) and CRP 156 mg/dl (31xULN) (Fig. 1A). Clinical (incl. urine analyses, blood culture) and radiological (CT scan of chest and abdomen, brain MRI) investigation, and FACS analysis of peripheral blood all showed no signs of infection or leukemia.

Multiplex cytokine analyses of three patient’s serum samples revealed a significant positive correlation between neutrophil count and G-CSF concentration (Fig. 1B). Additional proinflammatory (IL6, IFN $\gamma$, IL17, IL8,) and proangiogenic cytokines (VEGF, PDGF-bb), as well as immune stem cell growth factors (GM-CSF, MCSF), were elevated, as compared to the respective patients’ baseline concentration without neutrophilia. IL6 showed a remarkable increase of up to 17 × of baseline serum level (Fig. 1C).

To further identify patterns of cytokine differences, we performed unsupervised hierarchical clustering on normalized cytokine levels for patients 2 and 4. Increasing neutrophil counts clustered with an increase of IL2RA, IL8, PDGF-bb, IL17, and IFN$\gamma ,$ and elevation of CRP levels during PLR clustered with an increase of IL6 and VEGF (Fig. 1D).

## Discussion

Current state of research proposes the induction of PLR by cytokine secreting, highly aggressive tumors. The only therapeutic option is treatment of the underlying malignancy [3]. However, since most of the patients presented with rapid disease progression and lethal deterioration of their clinical status, fast-acting therapeutic approaches against the acute inflammatory reaction are needed.

PLR, as defined, was diagnosed in the reported patients 1, 3, 4 and 5 following thorough clinical investigation to rule out other causes for leukocytosis including infection and hematological malignancies. Hereby, neutrophils made up more than 90% of all measured leukocytes. Notably, neutrophilia was always accompanied by elevated CRP levels (Fig. 1A).

Serum cytokine analysis revealed a remarkable upregulation of IFN$\gamma$ and IL-6 which is associated with a concomitant increase of neutrophils count and CRP levels respectively (Fig. 1D).

The role of IFN$\gamma$ for antitumor immune responses is discussed controversially since both antitumorigenic and protumorigenic effects are described [7]. In the observed cases of PLR, serum IFN$\gamma$ upregulation is related to disease progression and worse patient outcomes, which proposes a protumorigenic role in this specific context.

IL-6 has been studied in autoimmune, acute inflammatory and malignant disease and assumed to be a prognostic biomarker for poor clinical outcomes [8]. The concomitant increase of IL6 and CRP in the here reported cases (Fig. 1D) is in concordance with progressive disease and onset of paraneoplastic neutrophilia. IL6 and other observed pro-inflammatory cytokines as well as the proangiogenic factor VEGF are involved in STAT3-regulated pathways and thus might contribute to tumor growth induction [9]. Further investigations are needed to reveal whether STAT3-involving pathways during PLR might present additional targets to inhibit disease progression.

Cytokine concentrations in the tumor and the periphery may vary significantly. Hence, further analysis of cytokine signatures in the tumor in comparison to serum levels during PLR is needed [10]. Targeting the mentioned cytokines by blocking antibodies or antibody-based cytokine delivery strategies experimentally can provide insights into their role in tumor control.

Not all observed cytokines are feasible therapeutic targets. The application of IL-6 antagonists was effective in several cases of acute inflammatory diseases including cytokine release syndrome [11, 12]. The combination of the anti-IL-6-antibody tocilizumab with immune checkpoint inhibition for the treatment of unresectable melanoma is currently being tested in a clinical trial (NCT03999749). Also depending on the results of this trial, targeting IL-6 might represent a clinically feasible treatment option, particularly for melanoma patients experiencing PLR.

## Supplementary Information

Below is the link to the electronic supplementary material.

Supplementary file1 (PDF 3911 KB)

## Data Availability

Detailed patient treatment history cannot be made available online due to privacy legislation in Germany. However, anonymized laboratory results can be provided following personal requests.

## Code availability

Available from corresponding author upon reasonable request.
