# Supplementary material for: Cytokine alterations during paraneoplastic neutrophilia and leukemoid reaction in patients with advanced melanoma
Source: Cancer Immunol Immunother. 2022 Jul 16;72(2):509–13. doi: 10.1007/s00262-022-03249-7 (PMC9870824; doi:10.1007/s00262-022-03249-7)
Supplement: Supplementary file 1 — Supplementary file1 (PDF 3911 KB) [file 262_2022_3249_MOESM1_ESM.pdf]

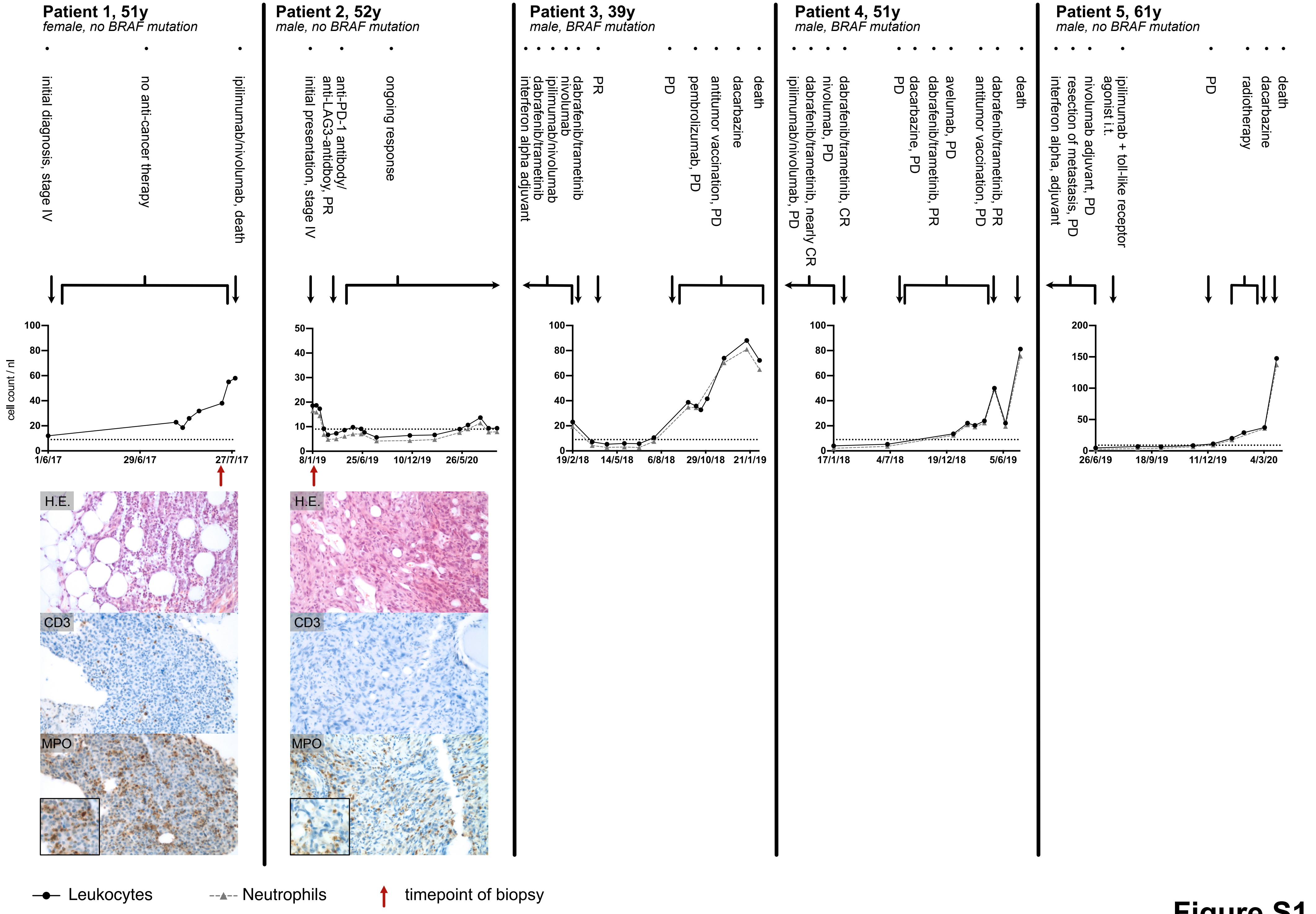

Patient 1, 51y
female, no BRAF mutation
initial diagnosis, stage IV
no anti-cancer therapy
ipilimumab/nivolumab, death
Patient 2, 52y
male, no BRAF mutation
initial presentation, stage IV
anti-PD-1 antibody/
anti-LAG3-antiboy, PR
ongoing response
Patient 3, 39y
male, BRAF mutation
interferon alpha adjuvant
dabrafenib/trametinib
ipilimumab/nivolumab
nivolumab
dabrafenib/trametinib
PR
PD
pembrolizumab, PD
antitumor vaccination, PD
dacarbazine
death
Patient 4, 51y
male, BRAF mutation
ipilimumab/nivolumab, PD
dabrafenib/trametinib, nearly CR
nivolumab, PD
dabrafenib/trametinib, CR
PD
dacarbazine, PD
avelumab, PD
dabrafenib/trametinib, PR
antitumor vaccination, PD
dabrafenib/trametinib, PR
death
Patient 5, 61y
male, no BRAF mutation
interferon alpha, adjuvant
resection of metastasis, PD
nivolumab adjuvant, PD
ipilimumab + toll-like receptor
agonist i.t.
PD
radiotherapy
dacarbazine
death
cell count / nl
1/6/17
29/6/17
27/7/17
8/1/19
25/6/19
10/12/19
26/5/20
19/2/18
14/5/18
6/8/18
29/10/18
21/1/19
17/1/18
4/7/18
19/12/18
5/6/19
26/6/19
18/9/19
11/12/19
4/3/20
H.E.
CD3
MPO
Leukocytes
Neutrophils
timepoint of biopsy

Figure S1

Figure S1 Scheme of treatment and histology Timeline of each patient, showing sequence and timepoints of each anti-cancer therapy in correlation with leucocyte count (round-dotted line) and neutrophil count (triangular-dotted line). Red arrows indicate time point of metastasis biopsy. Histological images of a metastasis during leukemoid reaction of patient 1 and patient 2 are shown. Upper image: 200x HE-morphological image. Middle image: 200x CD3-staining for lymphocytes. Lower image: 200x and 400x MPO staining of same metastasis, highlighting neutrophil granulocytes.
